# Blacking Boots

**Published:** 1858-01

**Authors:** 


### BLACKING BOOTS

Is a good morning's exercise; and it is certainly more creditable to have the covering for one's feet look neat and shiny every day, although done by ourselves, than to wear soiled boots from a false notion of self-respect. But suppose you have no blacking, nor the money to purchase it, even if it were to be had—what then? A naturally neat man never could imagine an excuse for dirt, so he would take any stiff brush that comes to hand, wet it a little in warm water, rub it on the bottom of any cooking utensil, then brush away on his boot until it shone almost like patent leather. We practiced this ourselves, over thirty years ago, when we had never heard of Day and Martin, or of patent leather.

